# Identification and functional analysis of *Tex11* and *Meig1* in spermatogenesis of *Hyriopsis cumingii*

**DOI:** 10.3389/fphys.2022.961773

**Published:** 2022-08-17

**Authors:** Yingduo Huo, Yang Gu, Mulian Cao, Yingrui Mao, Yayu Wang, Xiaoqiang Wang, Guiling Wang, Jiale Li

**Affiliations:** ^1^ Key Laboratory of Freshwater Aquatic Genetic Resources, Ministry of Agriculture and Rural Affairs, Shanghai Ocean University, Shanghai, China; ^2^ Shanghai Engineering Research Center of Aquaculture, Shanghai Ocean University, Shanghai, China; ^3^ Shanghai Collaborative Innovation for Aquatic Animal Genetics and Breeding, Shanghai Ocean University, Shanghai, China

**Keywords:** Hyriopsis cumingii, Tex11, Meig1, spermatogenesis, gonadal development, RNAi

## Abstract

**Abstract:** The process of spermatogenesis is complex and controlled by many genes. In mammals, *Testis-expressed gene 11 (Tex11)* and *meiosis expressed gene 1 (Meig1)* are typical spermatogenesis-related genes. In this study, we obtained the full length cDNAs for *Tex11* (3143bp) and *Meig1* (1649bp) in *Hyriopsis cumingii* by cloning*.* Among them, *Hc-Tex11* contains 930 amino acids and *Hc-Meig1* contains 91 amino acids. The protein molecular masses (MW) of *Hc-Tex11* and *Hc-Meig1* were 105.63 kDa and 10.95 kDa, respectively. Protein secondary structure analysis showed that Hc-TEX11 protein has three TPR domains. The expression of *Hc-Tex11* and *Hc-Meig1* in different tissues showed higher levels in testes. At different ages, the expression of *Hc-Tex11* and *Hc-Meig1* was higher levels in 3-year-old male mussels. During spermatogenesis, the mRNA levels of *Hc-Tex11*, *Hc-Meig1* gradually increased with the development of spermatogonia and reached a peak during sperm maturation. *Hc-Tex11* and *Hc-Meig1* mRNA signals were detected on spermatogonia and spermatocytes by *in situ* hybridization. In addition, RNA interference (RNAi) experiments of *Hc-Tex11* caused a down-regulated of *Dmrt1*, *KinaseX*, *Tra-2* and *Klhl10* genes and an up-regulated of *β-catenin* gene. Based on the above experimental results, it can be speculated that *Hc-Tex11* and *Hc-Meig1* are important in the development of the male gonadal and spermatogenesis in *H. cumingii*, which can provide important clues to better comprehend the molecular mechanism of *Tex11* and *Meig1* in regulating spermatogenesis of bivalves.

## Introduction

Reproduction is one of the most basic characteristics of living organisms, and most animals reproduce sexually. The process of spermatogenesis comprises mitosis, meiosis, and sperm deformation (Nishimura and L'Hernault, 2017). Many specific genes are involved in this process, and several genes involved in the spermatogenesis process have been identified in mammals, such as *Stra8, Cabs1 and Spag6* (Liu Y. H. et al., 2019; Yue et al., 2019; Zhang et al., 2021), and in fish, *Nanos1, Amh* and *Aqp1aa* (Li et al., 2015; Guo et al., 2017; Liang et al., 2020). However, studies on specific genes regulating spermatogenesis in mollusks are limited. It has been shown that *Klf4, Sox2, Sox17* in *Chlamys farreri* (Liang et al., 2017; Yang et al., 2017; Liang et al., 2019) and *Tssk1* in *Atrina pectinate* (Li H. H. et al., 2016) play a significant role in spermatogenesis^1^. In China, *Hyriopsis cumingii* is the most dominant freshwater pearl-cultivating mussel*,* and it accounts for more than 80% of the pearl-cultivating volume in captive freshwater mussels (Wang et al., 2014)^2^. The study found marked difference in pearl production and morphology between female and male mussels, with males being better than females (Wang et al., 2020). The gonads of freshwater mussels are of the follicular type^3^, consisting of three parts: follicles, genital canal and gonoduct, with the follicles and genital canal being the main parts that form germ cells (Chen and Shi, 2002; Pan et al., 2010). Heteromorphic chromosomes have not been identified in current studies on *H. cumingii*, so studies of sex and gametogenesis in *H. cumingii* revolve around the regulation of related genes (Wang et al., 2021a)^4^. Understanding the molecular mechanism of gametogenesis in *H. cumingii* can provide a basis for artificial propagation and hybrid breeding and is of great importance for seedling breeding^5^.

Direct homologs of testis-expressed (*Tex*) have been identified in vertebrates (mammals^6^, birds and reptiles), invertebrates and yeast (Bellil et al., 2021). *Testis expressed gene 11 (Tex11)* was originally identified as a germ cell-specific^7^, X-linked gene in mice (Yu et al., 2021)^8^. *Tex11*, also known as *Zip4h* in mice, is a direct mammalian homolog and a meiosis-specific protein in *Saccharomyces cerevisiae* and *Arabidopsis thaliana* that regulates the level of meiotic crossover (Wang et al., 2001). In mammals, *Tex11* is highly expressed specifically in the testis and localized to spermatocytes (Tang et al., 2011). *Tex11* competes with the estrogen receptor (ER), and when *Tex11* is overexpressed, it enhances the transcription of estrogen-response reporter genes but suppresses AKT and MAPK signaling pathways, resulting in reduced cell proliferation (Yu et al., 2012). In addition, some studies have shown that *Tex11* may be a key factor in germ cell development and ovarian in *Xenopus laevis* toads and affects fertility (Haselman et al., 2015). During meiosis, homologous chromosome pairing and recombination are required, a process that is closely linked to the activity of the synaptonemal complex (SC) (Heyting, 1996). Defects in meiosis can lead to haploid and polyploid organisms (Hassold and Hunt, 2001). *Tex11* is involved in the initiation of chromosomal synapses, and interacts with the central elements of SC, *Tex12* and *Sycp2*, in the formation of meiotic crossovers, providing a physical link between meiotic processes (Yang et al., 2008). *Tex11* genes have been studied more in mammals and less in aquatic animals. *Tex11* is an interesting factor in the late developmental stages of male *Anguilla* (Rozenfeld et al., 2019)*. Meiosis expressed gene 1* (*Meig1*) is a critical gene for the control of spermiogenesis and manchette structure. The *Meig1* defect destroys the head and tail of the sperm, impairing spermatogenesis and leading to infertility (Zhang et al., 2009). *Parkinson’s co-regulatory gene* (*PACRG*) interacts with *Meig1* to form a complex in the sperm tail that is essential for the transport of sperm flagellin and construction of sperm flagellum (Li W. et al., 2016). *Meig1* can also be used as a key gene for studying male sterility in *Vulpes fulvus* and *Alopex lagopus* hybrids (Yang et al., 2019). In *Acanthopagrus schlegelii*, *Meig1* transcript levels were higher in the testis than in the ovary (Zhang et al., 2019).

Due to the low evolutionary status of mollusks, the regulatory mechanisms of gonadal development and spermatogenesis are not yet clear. Bivalve mollusks contain multiple reproductive modes, including dioecism, hermaphroditic, and sex-reversed (Yue et al., 2020). This suggests that bivalves are suitable animals for studying gametogenesis and sex development. Some sex-linked genes such as *β-catenin, Dmrt1, Klhl10, KinaseX, Tra-2* have been identified in *H. cumingii*, and show expression characteristics of both sexes (Dong et al., 2020; Guo et al., 2020; Cui et al., 2021; Wang et al., 2021b). However, knowledge of the genes involved in spermatogenesis is still limited. *Tex11* and *Meig1* have been shown to be associated with mammalian spermatogenesis, while their role in invertebrate gametogenesis has not been reported. In this study, *Hc-Tex11* and *Hc-Meig1* were identified. The expression of *Hc-Tex11* and *Hc-Meig1* was analyzed in different tissues and at different periods. Additionally, the functions of *Hc-Tex11* and *Hc-Meig1* in gonads were analyzed by *in situ* hybridization and RNA interference (RNAi). Our data suggest that *Hc-Tex11* and *Hc-Meig1* are important for the spermatogenesis in *H. cumingii*. This provides a theoretical basis for the molecular mechanisms of spermatogenesis and sperm development in bivalves.

## Materials and methods

### Experimental material

Zhejiang Weiming aquaculture farm provided all the samples for this study. The collection and handling of *H. cumingii* was approved by the Institutional Animal Care and Use Committee (IACUC) of the Shanghai Ocean University, China.

Healthy 1, 2, and 3-year-old *H. cumingii* were brought back from the farm. The gonads were taken through a 1 ml microsyringe to differentiate gander under the microscope. The gonads, gills, kidneys, adductor, mantle, and liver were collected from 2-year-old *H. cumingii*. We collected gonadal tissues from the various stages of spermatogenesis (spermatogonia stage, spermatocyte stage, sperm maturation stage, sperm discharge stage, follicular atrophy stage). All samples were stored at 80°C for long-term storage, with three replicates per group.

### Extraction and reverse transcription of total RNA

The RNA was extracted by the Trizol, the purity of RNA was checked by NanoDrop 2000c (Thermo Scientific, US), followed by 1.0% agarose gel electrophoresis to check the integrity of RNA. cDNA synthesis according to PrimeScript^TM^ RT Reagent Kit with gDNA Eraser kit (TaKaRa, Japan), mixed in three parallel aliquots, and diluted 5-fold as a template for qRT-PCR.

### Full length cloning and sequence analysis of *Hc-Tex11* and *Hc-Meig1*

The sequences of *Hc-Tex11* and *Hc-Meig1* were obtained from the gonadal transcriptome database and were found to be incomplete at the 3′ end. Based on the original sequences, 3′ RACE primers for *Hc-Tex11* and *Hc-Meig1* were designed (Table 1). Reverse conversion to cDNA as template according to 3′-Full RACE Core Set with PrimeScript RTase kit. Rapid amplification was performed by nested PCR and cDNA ends. After PCR, the PCR products were ligated into pMD19-T vector, transformed into receptor *E. coli* DH5α, and selected white strains after blue-white spot and sent for testing.

**TABLE 1:** Primer names and sequences.

Primer name	Sequence (5′ to 3′)	Purpose
Tex11-1F	GAAGCCATTGCCACACTTAC	Validation
Tex11-1R	TGAAGGTTCTGTTCAGGGTTC	Validation
Tex11-2F	AGGGTGCCTTCCTGTCTATGTT	Validation
Tex11-2R	GCAGAGCTAGGATCAACCTTCT	Validation
Tex11-3F	TCGTGAATCCTTGGACTGGT	Validation
Tex11-3R	ATGGTCCAAAGACTCCTCAAGG	Validation
Tex11-4F	TCTCCAATGTGACAAGCACG	Validation
Tex11-4R	ATTCTTCAAGACTTCCCCGG	Validation
Meig1-F	TCACATCCACAGCCATCCAA	Validation
Meig1-R	TCATCTCTGTAGCCTGCCAACT	Validation
Tex11-3'	CAAGAAAGCACAGGGTCAATATCC	3' RACE
Meig1-3'	CCCCGACTTGCCACAACTCTAA	3' RACE
Q- Tex11-F	CCAATGCTAAGTTGCGAAAC	qRT-PCR
Q-Tex11-R	TCAGGGCAGTTACAATCTATCC	qRT-PCR
Q-Meig1-F	TCACATCCACAGCCATCCAA	qRT-PCR
Q-Meig1-R	TCATCTCTGTAGCCTGCCAACT	qRT-PCR
EFl-αF	GGAACTTCCCAGGCAGACTGTGC	Internal reference
EFl-αR	TCAAAACGGGCCGCAGAGAAT	Internal reference
ISH-Tex11-F	ATGCCTAACTCAGACCCCAA	ISH
ISH-Tex11-R	TAATACGACTCACTATAGGGGGAACAACTGGCTGCATTTG	ISH
ISH-Meig1-F	TCACATCCACAGCCATCCAA	ISH
ISH-Meig1-R	TAATACGACTCACTATAGGGTCATCTCTGTAGCCTGCCAACT	ISH
GFP-RNAi-F	TAATACGACTCACTATAGGGAAGGGCGAGGAGCTGTTCACCG	Negative control
GFP-RNAi-R	TAATACGACTCACTATAGGGCAGCAGGACCATGTGATCGCGC	Negative control
T-RNAi-F1	TAATACGACTCACTATAGGGTGCGGAAGATGTGTGACTGT	RNAi: G1
T-RNAi-R1	TAATACGACTCACTATAGGGGCTTGTCACATTGGAGAGCAA	RNAi: G1
T-RNAi-F2	TAATACGACTCACTATAGGGAGGGTGCCTTCCTGTCTATGTT	RNAi: G2
T-RNAi-R2	TAATACGACTCACTATAGGGTTTCCGATGTCGGGAGAGTT	RNAi: G2
Q-Dmrt1-F	GCTATTTCCAGAGGCCCAGA	RNAi
Q-Dmrt1-R	TGATGTCCGTGTCTCGTCAT	RNAi
Q-Tra2-F	TCACGAACTCCTTCCAGGAC	RNAi
Q-Tra2-R	CCTGGATCTCCTCCTCCTCT	RNAi
Q-KinaseX-F	CAAGCATGCAAGGATTTGCG	RNAi
Q-KinaseX-R	CCTGTGCTTAGTCTGGGTCA	RNAi
Q-Klhl10-F	TATGACGGCCATAACAGGCA	RNAi
Q-Klhl10-F	CGGCGTTATTCAAGCACTCA	RNAi
Q-β-catenin-F	CCAAGGTGGAGACCTGAACT	RNAi
Q-β-catenin-R	CCACTGGGTCATTCCCTGAT	RNAi

### Sequence analysis of *Hc-Tex11* and *Hc-Meig1*

The NCBI ORF Finder program was used to obtain open reading frame predictions of amino acid sequences; BLAST program was used to perform nucleotide and amino acid sequence similarity analysis of the obtained gene sequences with homologous species; SMART program predicts the secondary structure of proteins; SWISS-MODEL program predicts the tertiary structure; SignalP 4.1 Server program was used to predict signal peptides; the TMHMM Server v2.0 program was used to predict its transmembrane structure, and the NetPhos 3.1 program was used to discover its phosphorylation sites. The cloned cDNAs and amino acid sequences were analyzed by DNAMAN software, GeneDoc software performed multiple sequence alignment, and phylogenetic trees were constructed by MEGA 7.0 software using the neighbor-joining (NJ) method. Bootstrap was repeated 1,000 times to calculate the confidence values among the species.

### qRT-PCR

Real-time PCR primers for *Hc-Tex11* and *Hc-Meig1* were designed using Primer 5.0: Q-Tex11-F, Q-Tex11-R, Q-Meig1-F, Q-Meig1-R (Table 1). Using α subunit of *elongation factor 1* (*EF1-α)* (GenBank no. GW694601) as the reference (Bai et al., 2014), the reaction system (20 μL) as follows, 2×TB GreenTM Premix Ex Taq TM (Takara) 10 μL, primers 0.8 μL each, cDNA 1.6 μL, ddH_2_O 6.8 μL, three replicates of each sample. The calculation of relative expressions by the 2^−ΔΔCt^ method (Schmittgen and Livak, 2008). Significant differences were analyzed by SPSS18.0 software and plotted by SigmaPlot12.3.

### *In situ* hybridization

*In situ* hybridization primers were designed in the ORF region (Table 1), and the T7 promoter (5′-TAA​TAC​GAC​TCA​CTA​TAG​GG-3′) was added before the reverse primer. The T7 High Efficiency Transcription Kit (Transgen, China) was used for *in vitro* transcription. Gonadal tissues were fixed in 4% paraformaldehyde for 2 h, then paraffin embedded, cut to a thickness size of 6 μm and subjected to *in situ* hybridization using the Enhanced Sensitive ISH Assay Kit II (Boster, United States). Under a microscope, the hybridization signal was observed and photographed.

### RNAi assay

Two pairs of primers were designed using Primer 5.0 based on the *Hc-Tex11* cDNA sequence. Name the two interference strands as G1, G2 respectively. The primers of G1 were T-RNAi-F1 (5′-TAA​TAC​GAC​TCA​CTA​TAG​GGT​GCG​GAA​GAT​GTG​TGA​CTG​T-3′), T-RNAi-R1 (5′-TAA​TAC​GAC​TCA​CTA​TAG​GGG​CTT​GTC​ACA​TTG​GAG​AGC​AA-3′), amplifying a fragment of 393 bp. The primers of G2 were T-RNAi-F2 (5′-TAA​TAC​GAC​TCA​CTA​TAG​GGA​GGG​TGC​CTT​CCT​GTC​TAT​GTT-3′), T-RNAi-R2 (5′-TAA​TAC​GAC​TCA​CTA​TAG​GGT​TTC​CGA​TGT​CGG​GAG​AGT​T-3′), amplifying a fragment of 469 bp. The negative control dsRNA was a green fluorescent protein (GFP*)* sequence with no homology to *Hc-Tex11* (Table 1). The method for the synthesis step of dsRNA was referenced to that described by Wang (Wang et al., 2021b). Dilute to a certain concentration and store in the refrigerator at -80°C.

2-year-old male *H. cumingii* was divided into three groups (interference groups 1, 2, and negative control group), with six mussels in each group. Both dsGFP and two interference strands were diluted to 80 μg/μL, and 100 μL of *Hc-Tex11* dsRNA was injected into the adductor with a 1 ml microinjector, and tissues were collected after 48 h. RNA was extracted from the gonads, and the interference efficiency was detected and calculated by qRT-PCR. The expression of *Hc-Tra-2, Hc-Dmrt1, Hc-KinaseX, Hc-Klhl10* and *Hc-β-catenin* after dsRNA injection was also examined. The primer information is in Table 1.

### Statistical analysis

The experimental data were statistically analyzed using SPSS 18.0 software, and the data obtained were expressed as mean ± standard deviation. Significant differences were calculated using the independent samples *t*-test; *p* < 0.05 was considered statistically significant.

## Results

### Full length and amino acid sequence analysis of *Hc-Tex11* and *Hc-Meig1*.

**FIGURE 1:**
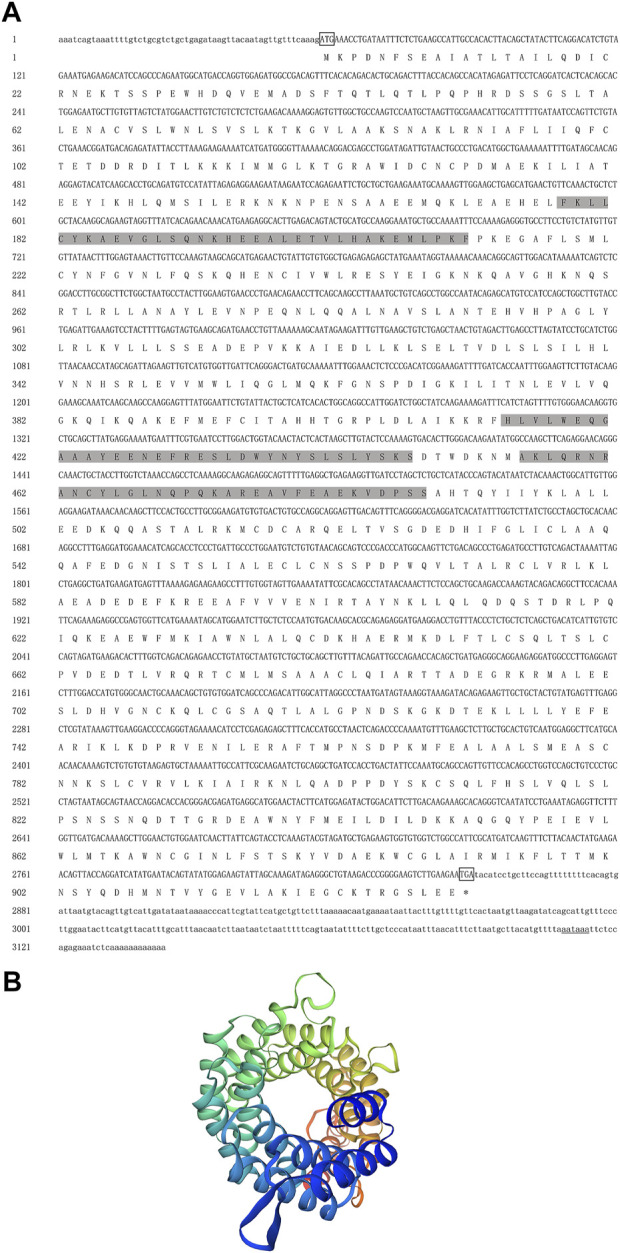
**(A)** Nucleotide and amino acid sequences of *Hc-Tex11.* Lowercase letters indicate 5′-UTR and 3′-UTR; start codon and stop codon are marked by boxes; plus-tail signals are underlined. The gray area is the TPR domain **(B)** SWISS-MODEL predicts the tertiary structure of Hc-TEX11.

**FIGURE 2:**
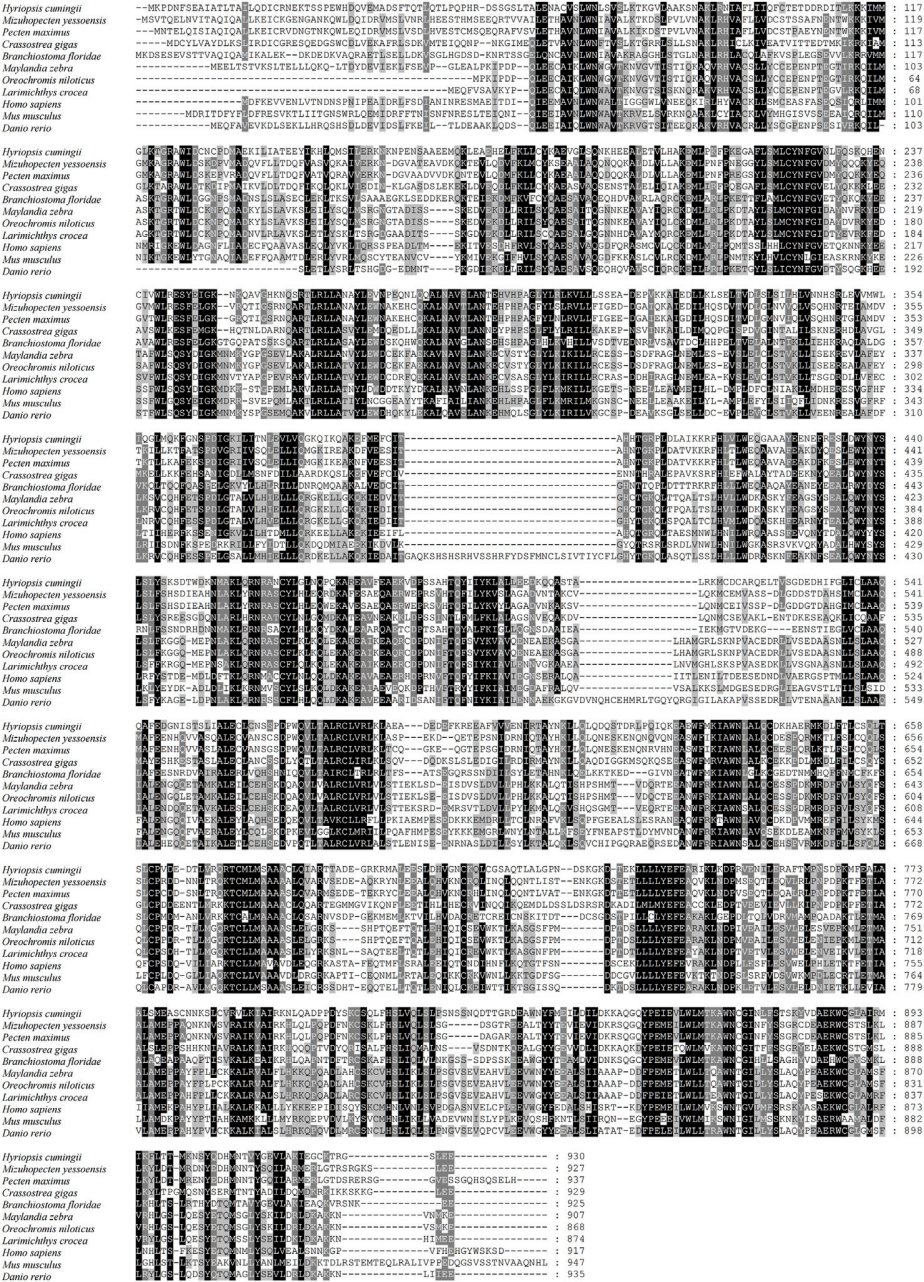
Multiple comparison of Hc-TEX11 amino acid sequence with other species. Black, same amino acid; grey, similar amino acid.

**FIGURE 3:**
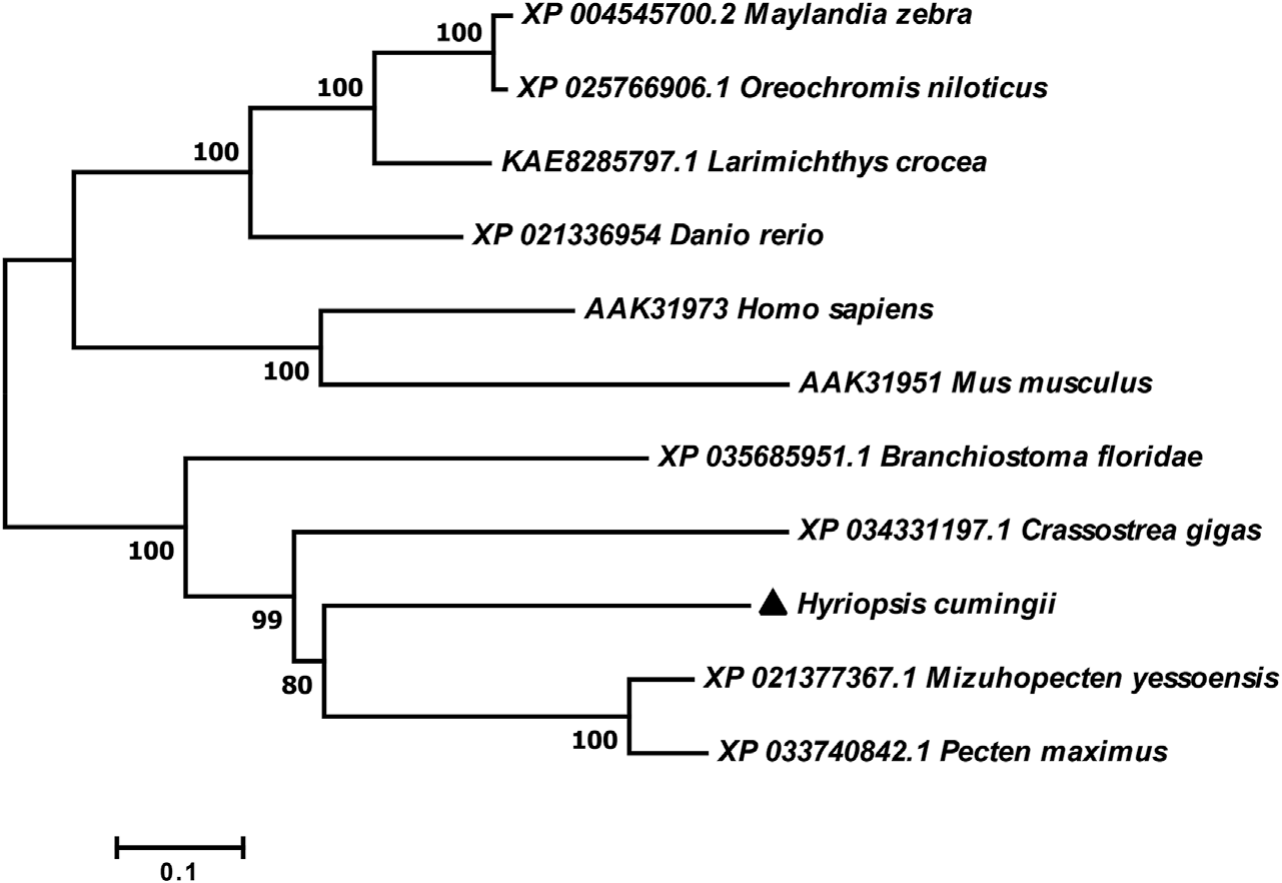
Phylogenetic tree of TEX11 in different species.

The full-length cDNA of *Hc-Tex11* gene was 3143 bp (GenBank no. ON804236), which includes 56 bp of 5′ untranslated region (5′-UTR) and 294 bp of 3′ untranslated region (3′-UTR) and 2793 bp of open reading frame (ORF), encoding 930 amino acids (Figure 1A). The protein predicted structure showed that it contains three tetrapeptide repeat proteins (TPR) (178-211aa, 414-447aa, and 455-488aa). The molecular weight (MW) of the protein was assessed to be approximately 105.63 kDa with a theoretical isoelectric point (PI) of 5.84. Phosphorylation site analysis revealed 38 serine (S) phosphorylation sites, 25 threonine (T) phosphorylation sites, and seven tyrosine (Y) phosphorylation sites. TMHMM predicted that the gene does not possess a transmembrane structure. SWISS-MODEL predicted the tertiary structure of the Hc-TEX11 protein (Figure 1B). The α-helix accounts for 70%, the irregular curl for 18% and no β-fold. Comparison of the amino acid sequence between *Hc-Tex11* and other species, the results showed that the similarity of *Hc-Tex11* among different species was high, including *Mizuhopecten yessoensis* (50.86%), *Pecten maximus* (50.23%), and *Crassostrea gigas* (45.57%)*.* The homology in humans and mice was 35.03% and 29.28%. Multiplex sequence analysis using GeneDoc revealed high amino acid similarity of *Hc-Tex11* with 10 other species (Figure 2). The phylogenetic tree was divided into two branches, vertebrate and invertebrate, in which the TEX11 protein of *H. cumingii* clusters into a branch with bivalve shellfish such as *M. yessoensis* and *P. maximus* (Figure 3). This result indicates that Hc-TEX11 is more closely related to mollusks and more distantly related to mammals, suggesting that the gene is relatively evolutionarily conserved.

**FIGURE 4:**
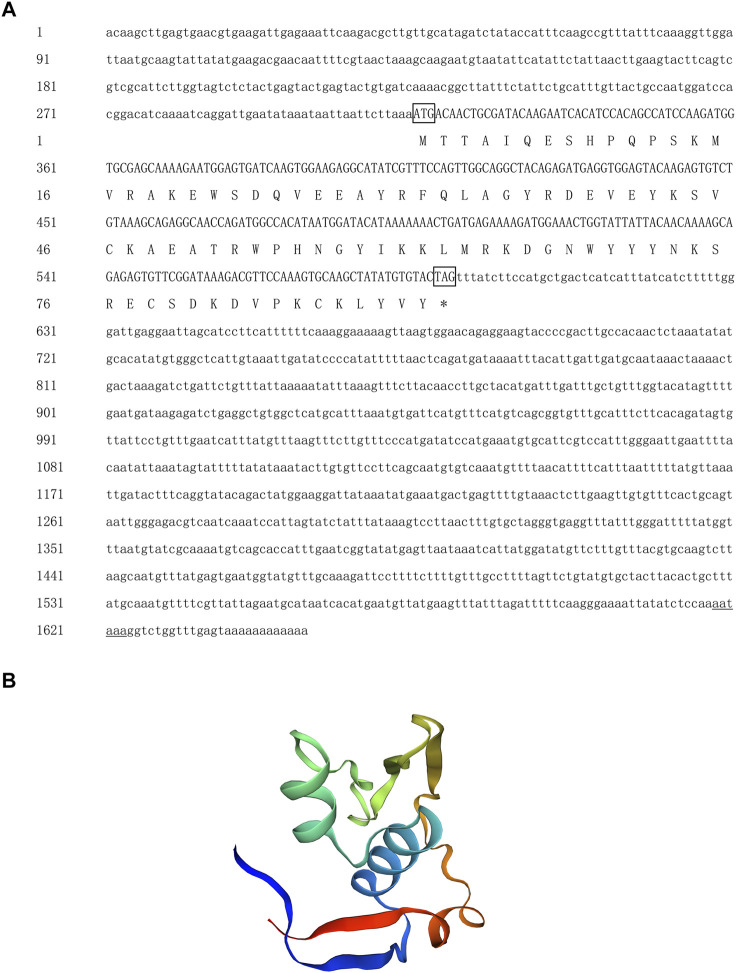
**(A)** Nucleotide and amino acid sequences of *Hc-Meig1*. Lowercase letters indicate 5′-UTR and 3′-UTR; start codon and stop codon are marked by boxes; plus-tail signals are underlined **(B)** SWISS-MODEL predicts the tertiary structure of Hc-MEIG1.

**FIGURE 5:**
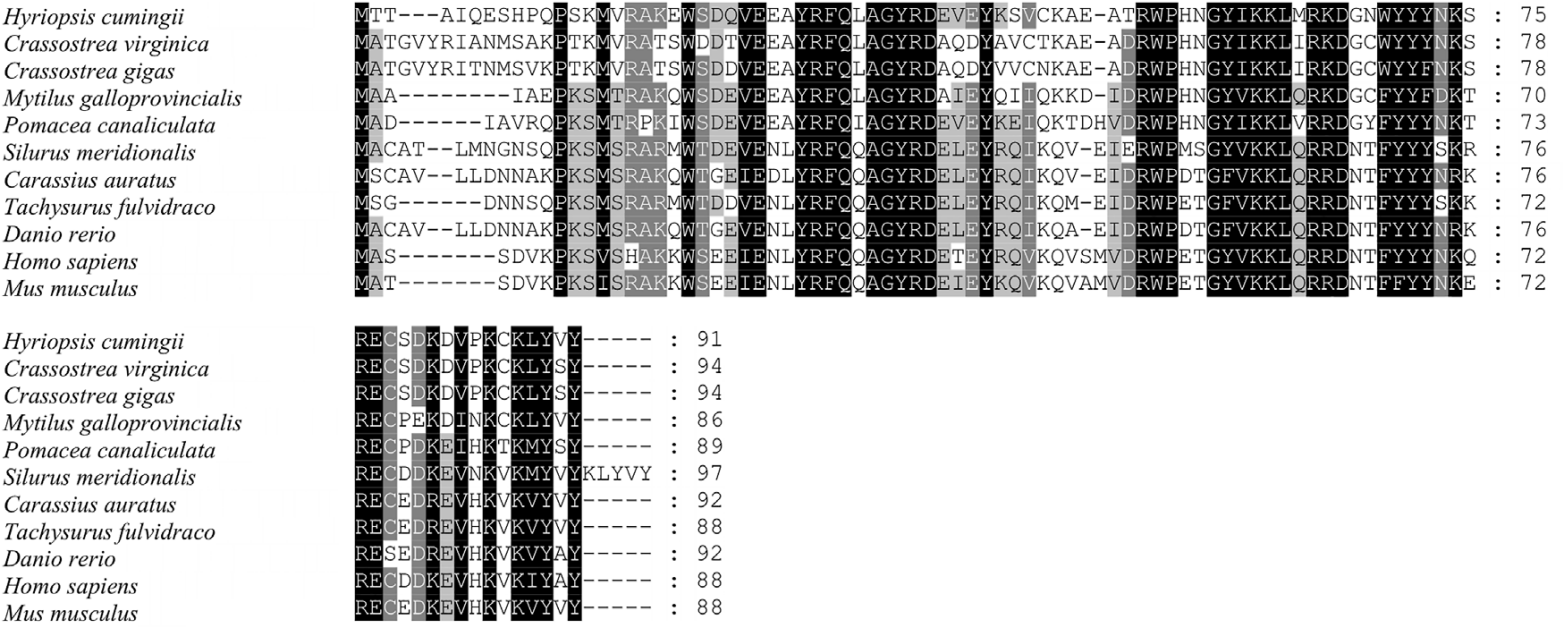
Multiple comparison of the Hc-MEIG1 amino acid sequence with other species. Black, same amino acid; grey, similar amino acid.

**FIGURE 6:**
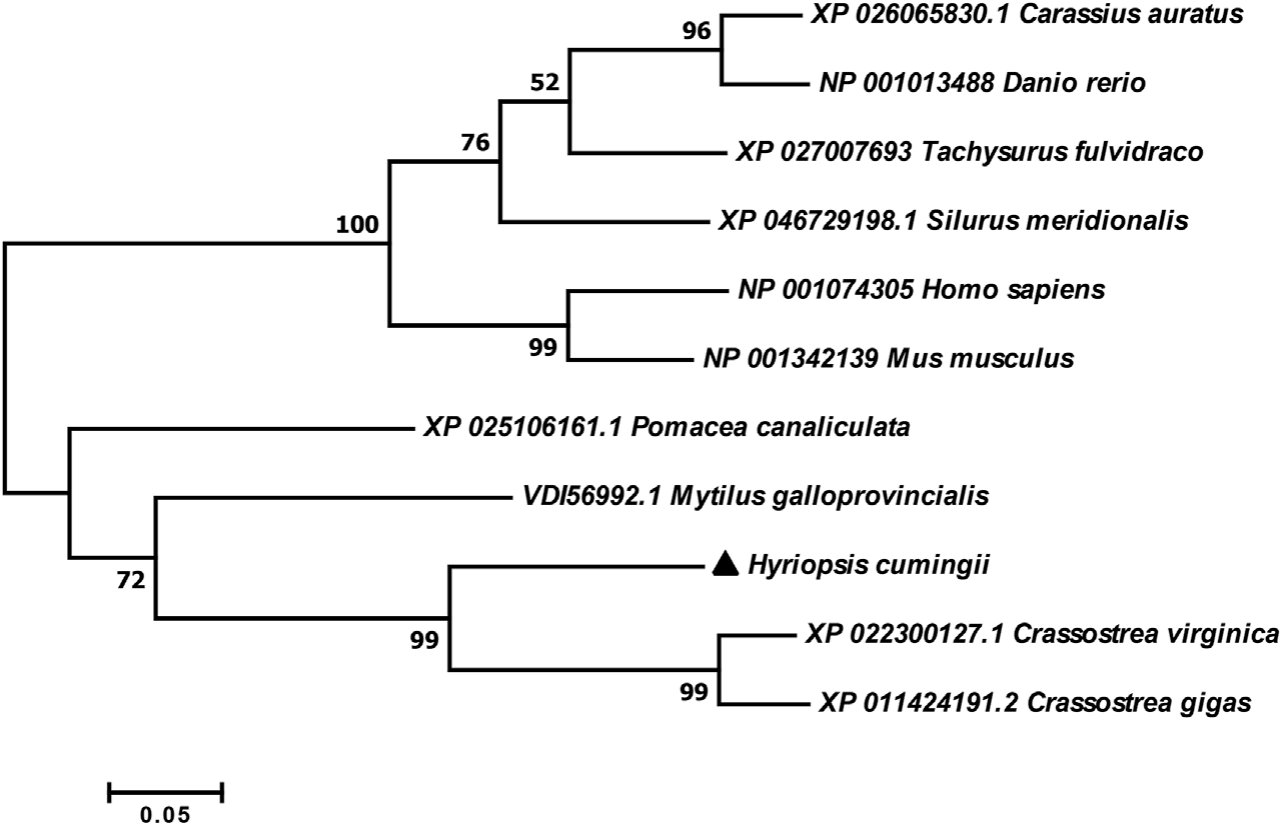
Phylogenetic tree of MEIG1 in different species.

The full length of the *Hc-Meig1* cDNA was 1649 bp (GenBank no. ON804237) with a 314 bp 5′-UTR, a 1059 bp 3′-UTR and a 276 bp ORF, encoding 91 amino acids (Figure 4A). *Hc-Meig1* has no transmembrane structure and secondary structure. The *Hc-Meig1* was 10.95 kDa and the PI was 8.94. Phosphorylation site analysis identified five serine (S) phosphorylation sites and two tyrosine (Y) phosphorylation sites. SWISS-MODEL predicted the tertiary structure of the Hc-MEIG1 protein (Figure 4B) with a QMEAN of -1.38, indicating a good match to the template protein. The α-helix accounted for 44%, the β-fold for 16%, and the irregular curl for 39%. Comparison of the amino acid sequence between *Hc-Meig1* and other species, the results showed that the similarity of *Hc-Meig1* among different species was high (66–74%), among which *Crassostrea virginica* (74.71%) was the highest. The homology in humans and mice was 52.94% and 56.47%. Multiple sequence analysis using GeneDoc revealed high amino acid similarity of *Hc-Meig1* with 10 other species (Figure 5). The phylogenetic tree was produced by MAGE 7.0 software, in which the MEIG1 protein of *H. cumingii* clusters into a branch with bivalve shellfish such as *C. virginica* and *C. gigas* (Figure 6). This result indicates that Hc-MEIG1 is more closely related to mollusks and more distantly related to mammals, suggesting that the gene is relatively evolutionarily conserved.

### Expression analysis of *Hc-Tex11* and *Hc-Meig1* genes in different tissues and periods.

**FIGURE 7:**
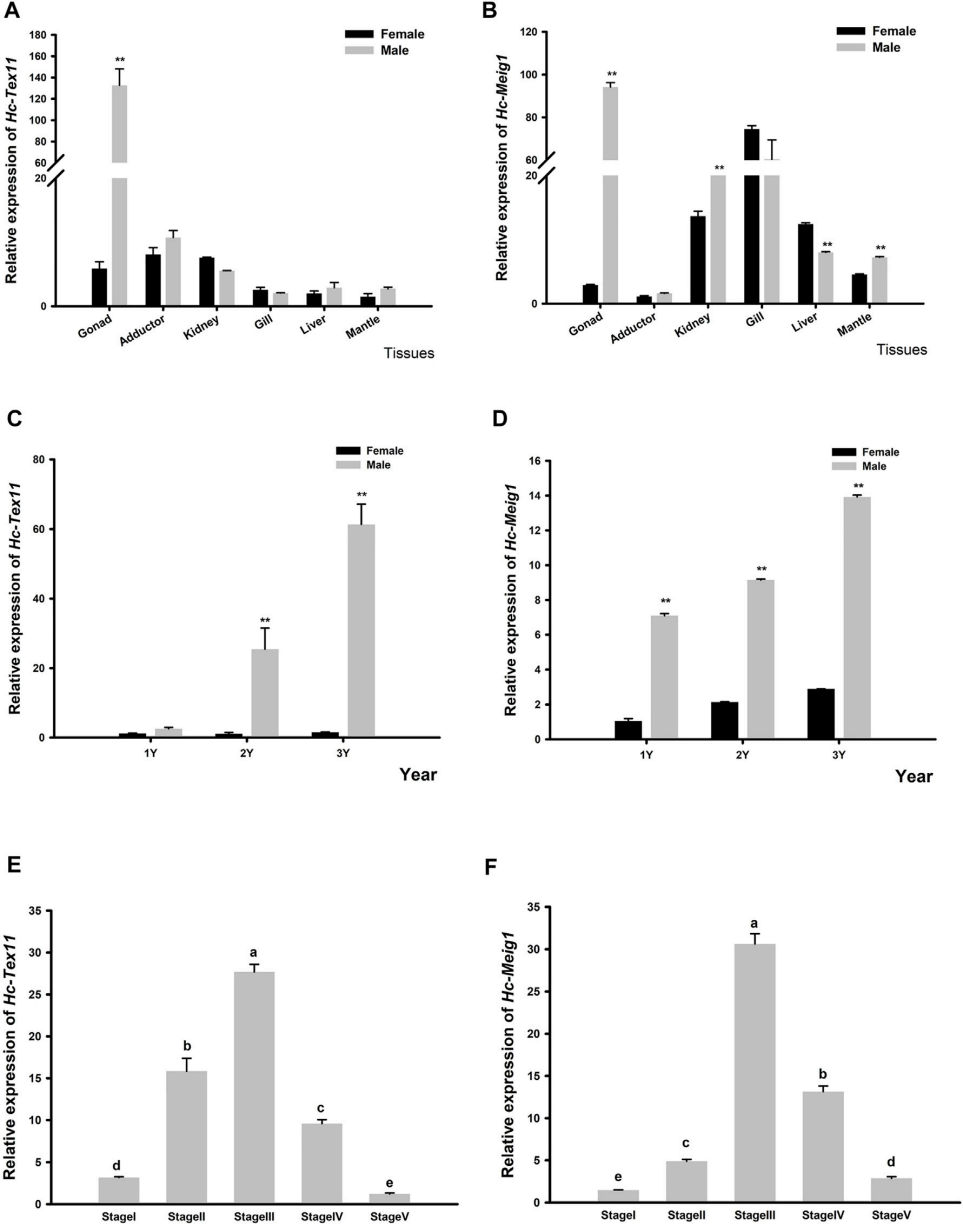
**(A)** Relative expression of *Hc-Tex11* gene in male and female tissue **(B)** Relative expression of *Hc-Meig1* gene in male and female tissue **(C)** Relative expression of the *Hc-Tex11* gene in 1-3-year-old male and female gonads **(D)** Relative expression of the *Hc-Meig1* gene in 1-3-year-old male and female gonads. Significant differences, * indicates *p* < 0.05, ** indicates *p* < 0.01 **(E)** Relative expression of *Hc-Tex11* gene during spermatogenesis **(F)** Relative expression of *Hc-Meig1* gene during spermatogenesis. Results are expressed as mean ± SD, and different letters **(A–E)** indicate statistically significant differences (*p* < 0.05). Stage1, spermatogonia stage; stage2, spermatocyte stage; stage3, sperm maturation stage; stage4, sperm discharge period; stage5, follicular atrophy stage.

The expression of *Hc-Tex11* and *Hc-Meig1* genes was detected in gonads, liver, gill, kidney, adductor, mantle. As well as the expression of male and female gonads at ages 1, two and three and spermatogenesis at each stage. The results showed that *Hc-Tex11* expression was highest in the testis and significantly higher than in other tissues (*p* < 0.01) (Figure 7A). The expression of *Hc-Tex11* increased with age in males, with the highest expression at 3 years of age and no significant change in expression in females (*p* < 0.01) (Figure 7C). During all stages of spermatogenesis, the expression level of *Hc-Tex11* gradually increases as spermatogonia continue to develop into sperm, reaching a maximum during the sperm maturation stage. A decreasing trend was observed from the sperm maturation stage to the follicular atrophy stage (*p* < 0.05) (Figure 7E).

The expression of *Hc-Meig1* was highest in the testes, followed by the gills, and the expression of the testes was significantly higher than that of the ovaries (*p* < 0.01) (Figure 7B). *Hc-Meig1* expression increases with age in both gonads, with higher expression in the testis than in the ovary at the same age (*p* < 0.01) (Figure 7D). In all stages of spermatogenesis, *Hc-Meig1* expression was significantly higher in sperm maturation than in other stages (*p* < 0.05) (Figure 7F). The expression pattern of *Hc-Meig1* in spermatogenesis is similar to that of *Hc-Tex11*.

### *In situ* hybridization of *Hc-Tex11* and *Hc-Meig1*

**FIGURE 8:**
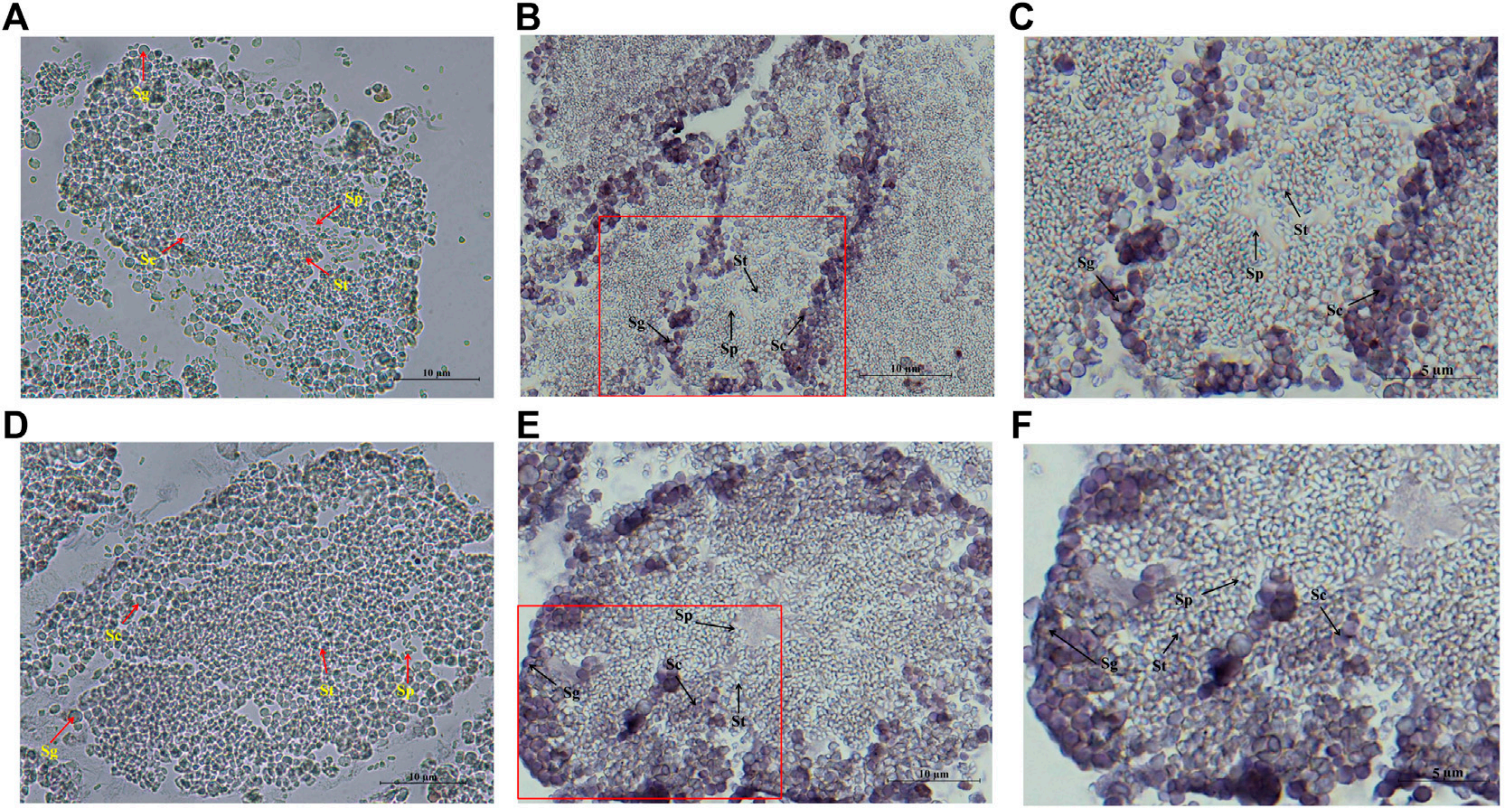
*In situ* hybridization of *Hc-Tex11* and *Hc-Meig1* genes in testes, **(A)** *Hc-Tex11* control group, **(B)** *Hc-Tex11* experimental group, **(C)** magnified image of *Hc-Te×11* experimental group, **(D)** *Hc-Meig1* control group, **(E)** *Hc-Meig1* experimental group, **(F)** magnified image of *Hc-Meig1* experimental group. Sg, spermatogonia; Sc, spermatocyte; St, spermatid; Sp, sperm.

The localization of *Hc-Tex11* and *Hc-Meig1* in the testis was detected by *in situ* hybridization, and male germ cells at different developmental stages (from spermatogonia to mature sperm) can be seen in the figure. *Hc-Tex11* (Figure 8B) and *Hc-Meig1* (Figure 8E) have distinct signals compared with the control group (Figures 8A,D). The results showed that the signals were located on spermatogonia and spermatocytes, whereas no signal was evident on spermatids and sperms.

### *Hc-Tex11* interference

**FIGURE 9:**
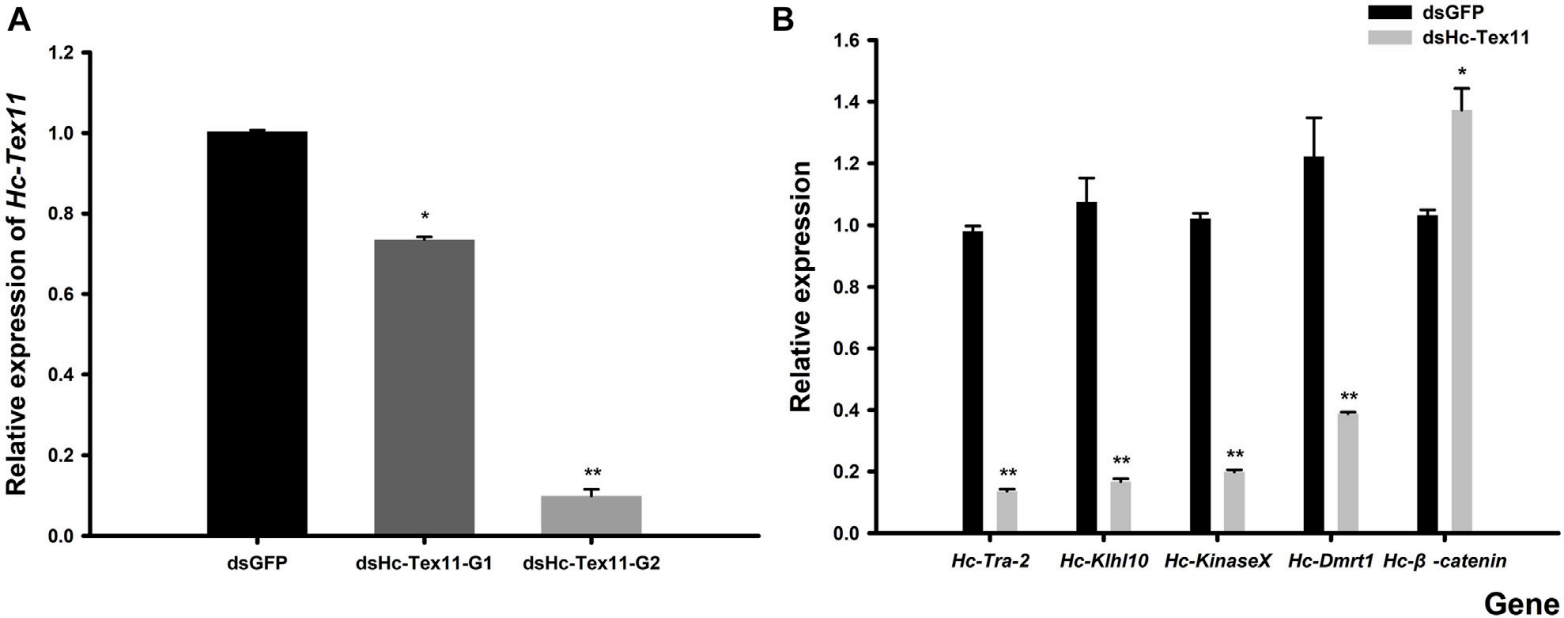
**(A)** Relative expression of *Hc-Tex11* gene in male gonadal tissue after RNAi **(B)** Effect of dsHc-Tex11-2 injection on the expression of *Hc-Tra-2, Hc-Klhl10, Hc-KinaseX and Hc-Dmrt1* and *Hc-β-catenin* genes in the testes of the *H. cumingii*. Control group, dsGFP; experimental group, dsHc-Tex11.

The expression of *Tex11* was measured in the male gonads of the *H. cumingii* after 48 h of interference. The results showed that the two interference strands synthesized in this experiment all played a role in the interference. The results of SPSS difference significance analysis showed that G1 was significantly different from the control group (*p* < 0.05) and G2 was highly different from the control group (*p* < 0.01). The silencing efficiency of the two interference strands was 26.74% and 90.13% (Figure 9A). And G2 had been selected as the interference strand for the subsequent experiments.

### Expression analysis of Hc-Tra-2, Hc-Klhl10, Hc-KinaseX, Hc-Dmrt1 and hc-β-catenin after RNAi

The silencing effect of dsHc-Tex11-G2 was 90.13%, which indicated that this interference strand was effective. Detect the effect of *Hc-Tex11* gene on other sex genes. Compared with the negative control group, the expression levels of *Hc-Tra-2, Hc-Klhl10, Hc-KinaseX* and *Hc-Dmrt1* were decreased, with interference efficiencies of 86.11%, 84.46%, 80.60% and 68.27% (*p* < 0.01), respectively, and the *Hc-β-catenin* expression level was significantly increased, 33.04% higher than that of the control group (*p* < 0.05) (Figure 9B).

## Discussion

In this study, the full length of *Hc-Tex11* was 3143bp, encoding 930 amino acids and containing three TPR domains (Figure 1A). The TPR domain usually forms a helix-turn-helix structure with internal gaps and contains 34 amino acids (Blatch and Lassle, 1999; Perez-Riba and Itzhaki, 2019). Proteins containing TPR domains are usually arranged in tandem 3–16 TPRs (Allan and Ratajczak, 2011). TPR structures are important in transcriptional regulation, protein kinase inhibition, peroxisomal protein and mitochondrial transport, cell cycle, immunity and viral replication, protein folding (Zhang et al., 2004; Liu et al., 2012; Rozenfeld et al., 2019). *Hsd-3.8, Klc3, Pp2ac* and *Ttc21a* genes possessing TPR domains were shown to be involved in mammalian spermatogenesis (Lin et al., 2002; Zhang et al., 2012; Pan et al., 2015; Liu W. J. et al., 2019). In aquatic animals, genes possessing the TPR domain also play an important role in the gonads. *Fem-1c* is involved in ovarian differentiation in *Danio rerio* (Shi et al., 2015), and *CypD* is highly expressed in the gonads of *H. cumingii* (Liu et al., 2017). Multiple sequence comparisons revealed that the structural domain of *Hc-Tex11* was more similar to that of mollusks compared to vertebrates, indicating that the gene has been relatively conserved during evolution. The full length of *Hc-Meig1* was 1649 bp (Figure 4A). The MEIG1 protein sequence of the *H. cumingii* was not found to have any known functional structure. However, Hc-MEIG1 has a high degree of amino acid sequence similarity to other species (Figure 5). It has been shown that the MEIG1 protein was phosphorylated *in vivo* and forms a dimer that enters the nucleus and binds to meiotic chromatin during the first meiotic division (ChenMoses et al., 1997; Steiner et al., 1999). Hc-MEIG1 contains seven phosphorylation sites, including serine and tyrosine. Multiple sequence comparisons showed that *Meig1* was highly conserved across species, implying that *Hc-Meig1* may function similarly to mammals in mollusks.

Up to now, the role of *Tex11* and *Meig1* genes in the testis and ovary has only been studied in mammals. *Tex11* is a testis-specific transcript that is required for chromosomal synapses, meiotic crossover and recombination, DNA double-strand break repair, and has been shown to be detected only in mammalian testes (Yu et al., 2021). In mature mice, *Meig1* is differentially expressed in females and males. Transcripts of *Meig1* are detected in pre-meiotic oocytes and mature testes, but not in mature ovaries (Don et al., 1994; Teves et al., 2013). In this study, *Hc-Tex11* was expressed in trace amounts in tissues other than the gonads, showing sexual dimorphism in the gonads and specific high expression in the testes (Figure 7A). This result is similar to the results of *Tex11* expression in pigs (Lin et al., 2002). *Hc-Meig1* was expressed in all the tissues examined, with the highest expression in the testis (Figure 7B). This is similar to the *Meig1_v1* results in the three *Meig1* transcripts in mice (Zhang et al., 2009). Expression in tissues other than gonads suggests that *Hc-Meig1* gene may also be involved in other functions in shellfish. *Hc-Tex11* and *Hc-Meig1* were most highly expressed in testes of 3 years old (Figures 7C,D). Based on the expression pattern, it is postulated that *Hc-Tex11* and *Hc-Meig1* are involved in the gonadal development of *H. cumingii*. All stages of the spermatogenesis were observed in male mussels above 2 years of age (Mi et al., 2002). Spermatogenesis is a cyclic process in which primordial germ cells undergo mitosis and meiosis to continuously form sperm, and mature sperm are gradually discharged until the follicle atrophy (Yu et al., 2008; Wang, 2010). In the spermatogenesis process of *H. cumingii*, the expression of *Hc-Tex11* and *Hc-Meig1* gradually increased during the development of spermatogonia to sperm and was highest during the sperm maturation stage (Figures 7E,F). This expression pattern suggests that *Hc-Tex11* and *Hc-Meig1* may be involved in spermatocyte meiosis like mammals. It is speculated that they play a potential role in the regulation of spermatogenesis in *H. cumingii*.

TEX11 protein was observed in mice from late spermatogonia onwards, with the highest levels in zygotene spermatocytes and the lowest levels in pachytene spermatocytes (Adelman and Petrini, 2008). *Meig1* was most abundantly expressed in pachytene spermatocytes (Don and Wolgemuth, 1992). In this study, mRNA signals of *Hc-Tex11, Hc-Meig1* were detected on both spermatogonia and spermatocytes of the testes (Figure 8). This result was similar to the results of mouse cell localization, further confirming the potential role of *Hc-Tex11* and *Hc-Meig1* in the spermatogenesis of the *H. cumingii*.

Several studies have shown that *Tex11* is strongly associated with azoospermia in human males (Sha et al., 2018; Yu et al., 2021). *Tex11* gene affects the quality of bull sperm and thus the early embryo, and deletion or mutation of the *Tex11* gene has been shown to cause meiotic arrest in animals (Yatsenko et al., 2015; Wu et al., 2020). In recent years, siRNA-mediated RNA interference has been widely used in the study of genes affecting spermatogenesis (Yang et al., 2020). In this study, *Hc-Tex11* was silenced by RNAi to further explore the role of *Hc-Tex11* in the spermatogenesis of *H. cumingii* (Figure 9A). To further investigate the effect of *Hc-Tex11* deficiency on gonadal development, we examined the expression levels of several genes that have been shown to play evolutionarily conserved roles in gonadal development in the *H. cumingii*, including *Hc-Dmrt1, Hc-β-catenin, Hc-KinaseX, Hc-Tra-2,* and *Hc-Klhl10*. The expression of *Hc-Dmrt1* and *Hc-Tra-2* genes, which are important in sex determination and differentiation, and early gonadal development (Kim et al., 2003; Erickson and Quintero, 2007; Wang et al., 2019), was down-regulated following knockdown of *Hc-Tex11*. *Dmrt1* maintains male germ cell differentiation and low expression results in impaired gonadal differentiation (Guo et al., 2020). As a result, it is speculated that *Hc-Tex11* may be involved in gonadal development and germ cell differentiation in *H. cumingii,* by regulating the expression of *Hc-Dmrt1* and *Hc-Tra-2*. *KinaseX* and *Klhl10* genes are involved in spermatogenesis, sperm capacitation and fertilization (Stogios et al., 2005; Dong et al., 2020; Cui et al., 2021). The expression of *Hc-KinaseX* and *Hc-Klhl10* was down-regulated after *Hc-Tex11* knockdown. It is speculated that *Hc-Tex11* may be involved in the spermatogenesis of *H. cumingii* by regulating the *Hc-KinaseX* and *Hc-Klhl10*. *β-catenin* is a key factor for pro-ovarian and anti-testicular whose activation prevents testis development (Li et al., 2014; Wang et al., 2019). The female-associated gene *Hc-β-catenin* was up-regulated after knockdown of *Hc-Tex11*, suggesting a possible negative regulatory relationship between *Hc-Tex11* and *Hc-β-catenin* (Figure 9B). In summary, there is a link between the *Hc-Tex11* and sex-related genes, but their interrelationships and the mechanisms regulating spermatogenesis need further research to be explored.

## Conclusion

In conclusion, in this study, we obtained the full length cDNAs of *Hc-Tex11* and *Hc-Meig1* by cloning. *Hc-Tex11* and *Hc-Meig1* play a significant role in spermatogenesis and male gonad development in *H. cumingii*, as shown by qRT-PCR and *in situ* hybridization experiments, and revealed that their cellular localization was on spermatogonia and spermatocytes. The RNAi results illustrate some association between *Hc-Tex11* and sex-related genes in *H. cumingii*. Among them, *Hc-Dmrt1, Hc-KinaseX, HcTra-2* and *Hc-Klhl10* were down-regulated and *Hc-β-catenin* was up-regulated. It shows that *Hc-Tex11* regulates gonadal development, but this regulation needs to be further investigated.

## Data Availability

The raw data supporting the conclusions of this article will be made available by the authors, without undue reservation.

## Footnotes

1
https://www.ncbi.nlm.nih.gov/orffinder/

2
https://blast.ncbi.nlm.nih.gov/Blast.cgi.

3
http://smart.embl-heidelberg.de/

4
https://swissmodel.expasy.org/

5
http://www.cbs.dtu.dk/services/TMHMM/

6
http://www.cbs.dtu.dk/services/SignalP/

7
https://web.expasy.org/protparam/

8
http://www.cbs.dtu.dk/services/NetPhos/

## Abbreviations

Tex11 Testis-expressed gene 11; Meig1 Meiosis expressed gene one; β-catenin beta-catenin; Dmrt1 Doublesex and Mab-3 Related Transcription Factor 1; Klhl10 Kelch-like protein 10; KinaseX protein kinaseX gene Tra-2 Transformer-2; TPR tetrapeptide repeat proteins.
